# Age Related Assessment of Sugar and Protein Intake of *Ceratitis capitata* in *ad libitum* Conditions and Modeling Its Relation to Reproduction

**DOI:** 10.3389/fphys.2017.00271

**Published:** 2017-05-08

**Authors:** Nikos A. Kouloussis, Petros T. Damos, Charalambos S. Ioannou, Constantinos Tsitsoulas, Nikos T. Papadopoulos, David Nestel, Dimitris S. Koveos

**Affiliations:** ^1^Laboratory of Applied Zoology and Parasitology, School of Agriculture, Aristotle University of ThessalonikiThessaloniki, Greece; ^2^Department of Agriculture Crop Production and Rural Environment, University of ThessalyVolos, Greece; ^3^Institute of Plant Protection, Agricultural Research Organization (ARO), Volcani CenterBet Dagan, Israel

**Keywords:** life time feeding, age related reproduction, Lorentz distribution, capillary feeding, individual medflies

## Abstract

In the inquiry on the age related dietary assessment of an organism, knowledge of the distributional patterns of food intake throughout the entire life span is very important, however, age related nutritional studies often lack robust feeding quantification methods due to their limitations in obtaining short-term food-intake measurements. In this study, we developed and standardized a capillary method allowing precise life-time measurements of food consumption by individual adult medflies, *Ceratitis capitata* (Diptera: Tephritidae), under laboratory conditions. Protein or sugar solutions were offered via capillaries to individual adults for a 5 h interval daily and their consumption was measured, while individuals had lifetime *ad libitum* access to sugar or protein, respectively, in solid form. Daily egg production was also measured. The multivariate data-set (i.e., the age-dependent variations in the amount of sugar and protein ingestion and their relation to egg production) was analyzed using event history charts and 3D interpolation models. Maximum sugar intake was recorded early in adult life; afterwards, ingestion progressively dropped. On the other hand, maximum levels of protein intake were observed at mid-ages; consumption during early and late adult ages was kept at constant levels. During the first 30 days of age, type of diet and sex significantly contributed to the observed difference in diet intake while number of laid eggs varied independently. Male and female adult longevity was differentially affected by diet: protein ingestion extended the lifespan, especially, of males. Smooth surface models revealed a significant relationship between the age dependent dietary intake and reproduction. Both sugar and protein related egg-production have a bell-shaped relationship, and the association between protein and egg-production is better described by a 3D Lorenzian function. Additionally, the proposed 3D interpolation models produced good estimates of egg production and diet intake as affected by age, providing us with a reliable multivariate analytical tool to model nutritional trends in insects, and other organisms, and their effect upon life history traits. The modeling also strengthened the knowledge that egg production is closely related to protein consumption, as suggested by the shape of the medfly reproduction-response function and its functional relationship to diet intake and age.

## Introduction

During the past, considerable attention has been devoted to the analysis of food consumption and appetite on groups of animals or on individual organisms (Kogan and Parra, 1981; Cullison, 1982; Gomez-Amaro et al., 2015). However, most of these studies have focused on the total amount of food consumed by the individuals throughout a period or until death, and not at the daily feeding events and individual caloric consumption (Ja et al., 2007). That is, the dissection of ingestion into specific meal parameters such as meal-size and frequency (i.e., prandiology), has been poorly addressed. This level of resolution is in fact of importance in biomedical studies that aim at understanding the physiology and regulation of appetite (Ja et al., 2007). Moreover, from an ecological and demographic perspective, the quantification of diet-size, and frequency of meals through the lifetime of the organism may provide information on changes in feeding patterns throughout aging, allowing us to generate hypothesis on the mechanisms involved in senescence and management of metabolic energy.

Several lines of research on food consumption, physiology, metabolism, and pathology use model-organisms to answer questions with practical implications to human nutrition and disease. Classical model organisms for answering medical questions include laboratory rats and insects. In insects, the vinegar fly, *Drosophila melanogaster—*ironically known as the entomological laboratory “rat”—is mostly used to answer genomic and nutrigenomic questions related to metabolism and diseases (Bharucha, 2009; Ashburner and Bergman, 2016; Ugur et al., 2016). Other insects, like fruit flies (Tephritidae), have also been used as model organisms, but especially in the inquiry of demographic and actuarial questions (Carey and Papadopoulos, 2005). The advantage in the use of insects as model-organisms is related to their short generation time, ability to easily produce large quantities of organisms in a very short period, relative simplicity in their genetic manipulation and development of genetic-lines and close similarity with metabolic pathways of vertebrates, relatively low-costs, and minimal ethical questions of experimentation (Schneider, 2000). Due to these traits, insects can serve as excellent model-organisms to explore questions of nutrition and prandial behavior. Prandiology, refers to the study of specific parameters such as the size and frequency of meals (see Ja et al., 2007).

Methodologies to measure individual intake of nutritional solutions by insects have been proposed. These include the Capillary Feeder (CAFE) method (Ja et al., 2007) used with *D. melanogaster*, the Phagostimulation Unit Bioassay (PUB; Nestel et al., 2004) used with tephritid fruit flies, and the use of pipette tips as dispensers of food solution used also with tephritids (Fanson et al., 2009). These methods have been used to answer questions such as the toxic effect and kinetics of ingesting stomach poison in fruit flies (Nestel et al., 2004), the prandiology of *D. melanogaster* (Ja et al., 2007), the effects of the “social context” on intake and energy management in tephritids (Zur et al., 2009) and the effect of dietary restriction on reproduction and survival of Tephritidae fruit flies (Fanson et al., 2009). An interesting aspect of prandiology investigation is the possibility of linking individual intake with physiological performance, reproduction, and longevity. While Fanson et al. (2009) linked intake with reproduction and survival on the Queensland fruit fly, their system provided intake information at a 4 day intervals. On the other hand, the PUB system has technical limitations to be able to concomitantly measure intake and reproduction. The only alternative to be able to provide this type of information is the use of the CAFE system with the specific aim of also measuring individual nutrition and reproductive effects. The intentions of the present study were, thus, to explore the use of CAFE as a prandiology system suitable for providing information on individual daily intake of nutrients and its effect on reproduction and longevity. We used the Mediterranean fruit fly (medfly), *Ceratitis capitata* (Diptera: Tephritidae), as a model organism to demonstrate the ability of the CAFE system to provide prandiology information at a resolution of the individual organism and at relatively short time intervals. Former quantitative methods have focused on measuring feeding of insects on the simultaneous combinations of different ratios of protein and sugar (Nestel et al., 2016). In the current study, however, one source of food is given *ad libitum* and in solid form, while the other is given for a specific time-period in liquid form and measured, gaining discrete measurements on the consumed amount of liquid food source in the presence of a second nutrient source. We also show the ability of our system to provide individual information on reproduction, longevity, and nutrition, and mathematically and statistically link between the different measured factors at the individual level. Our objectives were: to develop and standardize a reliable method for prandiology studies in fruit flies and to apply modern multivariate analysis techniques to the results, ultimately providing a useful framework for future prandiology studies.

## Materials and methods

### Experimental flies and laboratory conditions

The medflies used in the experiments were of the F_2_–F_8_ laboratory generation established from pupae collected from naturally-infested figs (*Ficus carica* L.), pears (*Pyrus communis* L.), apricots (*Prunus armeniaca* L.), and peaches (*P. persica* L.) around Thessaloniki (northern Greece, 40°31′N, 22°58′E). Specifically, we used flies of the F2–F6 laboratory generations for standardizing the methodology (see below), whereas the main life time experiments were performed using adults of the F7–F8 generations. Collected pupae were mixed to create batches of ~300 individuals each and placed in 9 cm diameter plastic Petri dishes until adult emergence. The colonies were maintained in wooden, nylon screened, holding cages (30 by 30 by 30 cm) and provided with food (a mixture of yeast hydrolysate and sugar in a ratio of 1:4 in the form of solidified droplets), water and oviposition substrates. Each oviposition substrate consisted of a red, plastic, hollow hemisphere (5 cm diameter) with about 100 evenly distributed holes of 0.7 mm diameter through which females laid their eggs. The base of each dome was fitted into a hole made on the lid of a plastic Petri dish. To stimulate oviposition, 5 mL of freshly-squeezed orange juice were placed into the Petri dish. Eggs were collected with a fine brush and placed on standard larval medium for immature development. Details regarding the composition as well as the preparation procedures of larval diet are described by Boller (1985). The experiments were conducted in the laboratory of Applied Zoology and Parasitology of the Aristotle University of Thessaloniki, Greece, under a 14:10 h light: dark cycle at 25 ± 2°C and 55 ± 10% relative humidity.

### General methodology to assess food ingestion and Egg-laying

**Figure 1:**
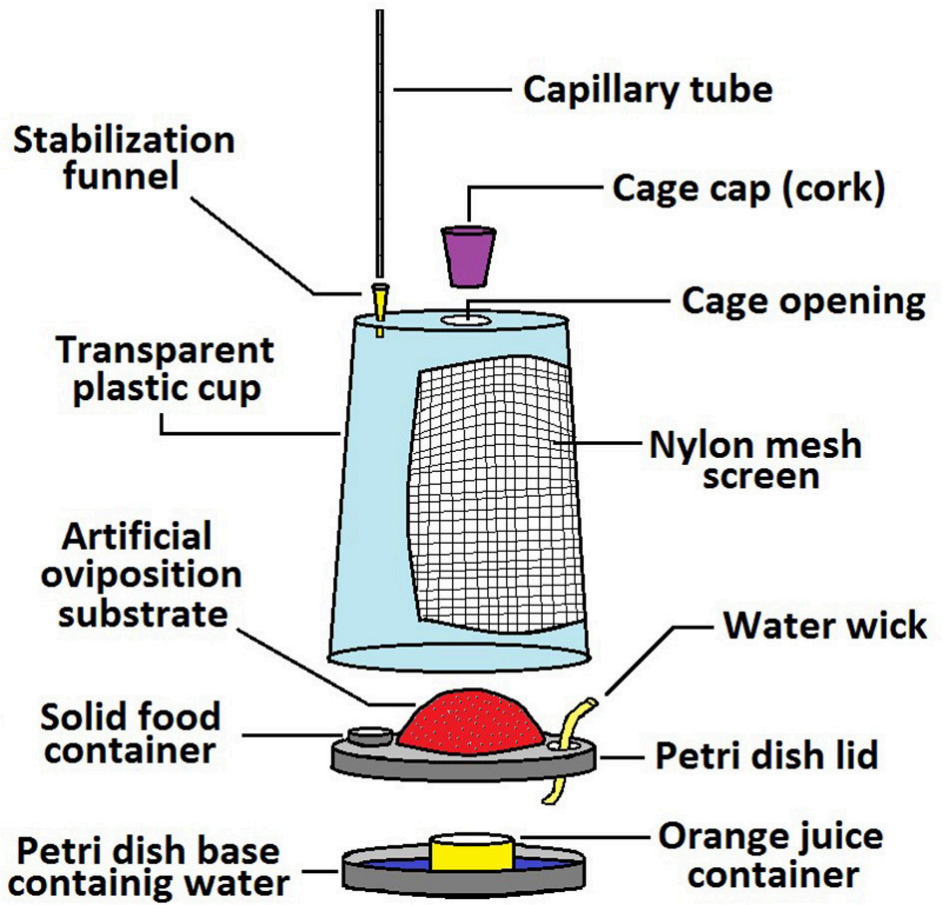
**Schematic diagram of the individual cage and capillary tube used to measure the lifetime daily intake of liquid food and oviposition by *C. capitata***. The cage consisted of an inverted transparent 400 ml volume plastic drinking cup (12 cm high, 6.5 cm base diameter, and 9 cm top diameter). The bottom part of the cage was the lid of a 9 cm-diameter plastic Petri dish attached to the open large surface of the inverted cup. A lateral 5 × 8 cm window covered with nylon-mesh-screen was perforated on the cup's side for ventilation while a 2 cm-diameter opening was cut on cup's top center serving as an entrance for the adult medflies and a cork was used as a cap. Sugar or protein hydrolysate was provided as a water solution by the capillary tube at the top of the cage whereas solid food was provided in a small container at the base of the cage. Peripherally of the top smaller surface of the inverted plastic cup (2–3 mm from the edge) a hole was made and a cut pipette tip (3 cm height) was set stabilizing the vertically inserted glass capillary tube. The bottom 1 mm end of the capillary projected inside the cage. To decrease evaporation, the top part of the capillary was sealed with a tiny drop of paraffin oil. A 5 cm diameter circular hole was cut in the middle of the petri dish lid forming the base of the cage and a hollowed plastic dome perforated with small holes serving as an oviposition substrate was attached. The petri dish base under the dome contained water and a smaller cylindrical cup filled with 5 ml of fresh orange juice (plus 1% sorbic acid) serving as an oviposition stimulant. The orange juice was replaced every 2 days. Individual cages for males lacked both the oviposition substrate and the orange juice container.

The method we developed is a modification of the CAFE bioassay proposed by Ja et al. (2007). Medflies were placed inside 400 ml volume individual plastic cages. Tested solutions were provided in a glass capillary tube (Cat. No. 29 000 00; Marienfeld-Superior, Germany), with one end of the capillary inserted inside the cage and accessible to the flies. Water was provided *ad libitum*. In addition to water, we placed complementary solid food inside the cage. Solid food consisted of either sugar, if we were measuring solutions of protein hydrolysate in the capillary, or solid protein hydrolysate, if we were measuring solutions of sucrose in the capillary. This experiment allowed quantification of ingestion of a nutrient while the other nutrient was provided *ad libitum*. In the experiments where oviposition was measured, the cages also included a device for egg-laying, which allowed recording of the amount of eggs laid during the experimental period. A detailed drawing of the cage and the capillary feeding mechanism is shown in Figure 1.

Prior experimental experience with the CAFE method has shown that to achieve reliable and high precision measurements it is required a low evaporation through the capillaries providing the food solution. Therefore, it is necessary to sustain a rather humid environment during the time capillaries are inserted in the cages or otherwise evaporation of capillary solution is relatively high. In our experiments, to reduce evaporation and prevent dripping, and thus increase the accuracy of our method, a small quantity of paraffin oil was added in the upper extreme of the capillary tube. In addition to that we conducted the experiments inside the “Humidity Preservation Chamber” (HPC) described in the next paragraph. In each HPC we established five individual cages, plus a control cage which did not contain any fly and served to measure evaporation from the capillaries. The descent of the meniscus of the food solution in the capillaries as the insects were feeding allowed continuous, unambiguous measurement of feeding events and diet consumption. This measurement was corrected by subtracting the amount evaporated from the capillary in the control cage. The remaining amount was, thus, recorded as the volume of solution consumed by an insect during the 5 h of exposure.

### Effect of the high relative humidity on Egg production

It is known that high humidity levels may affect oogenesis in fruit flies (Broufas et al., 2009). To assess a possible negative effect of the required high relative humidity in our method on egg production, we conducted an additional experiment. Females were placed in four 25 by 25 by 35 cm cages in groups of 125 per cage. The cages contained water and ample sugar and protein (mixed in a ratio of 3:1, respectively) in solid form. In two of these large cages (referred to as HPCs), relative humidity was maintained high for 5 h every day (from 12:00 to 17:00 h) using water soaked Wettex and plastic that covered the screen openings. The other two cages remained uncovered serving as controls; relative humidity in these cages was low, around 40%, and at the same level as in the experimental room. Daily, and for eight consecutive days, 10 females from each of the two HPCs and the control cages, totaling 160 females for the treatment and 160 for the control, were sacrificed in alcohol and their ovaries dissected to assess the number of mature eggs.

### Effect of nutrient concentration on food intake and oviposition

To select a concentration of liquid food and to “calibrate” our method we used only females. We tested three different concentrations (5, 10, or 20% w/v) of liquid sugar and liquid protein. When testing liquid sugar, protein was provided *ad libitum*, whereas, when testing liquid protein solid sucrose was provided *ad libitum*. We used 20 individual female cages per concentration. Five individual cages hosting females, and one control cage without a female serving to measure evaporation, were placed inside an HPC. During 15 days, food was provided to these females for 5 h per day, from 12:00 to 17:00 h, and consumption was measured at the end of the 5 h period. The aim of the experiment was to find a concentration that corresponded to a volume of consumption large enough to be measured reliably, while also providing the females with adequate nutrients to mature their eggs. To measure the reproductive potential associated with diet type, after 15 days all females from the different treatment concentrations were sacrificed in alcohol and their ovaries were dissected to quantify mature eggs.

### Life time consumption patterns of protein and sugar, and related Egg-laying patterns

The previous experiments provided us with the basis to establish a long-term experiment to measure food intake in male and female medflies and female egg-laying patterns. Males and females were maintained individually (as described above) in the individual cages placed inside HPCs. Thirty individual males were exposed for 5 h daily to a solution of 20% sucrose in capillaries to measure daily intake, while they were allowed free access to protein hydrolysate *ad libitum*. Simultaneously, 30 individual cages with single males were exposed to the opposite condition: 20% protein hydrolysate solution provided to them in capillaries for 5 h. daily, while sucrose was provided *ad libitum*. Simultaneously, two groups of 30 individual female cages were also established in a similar fashion to the ones described for males, and intake of 20% protein hydrolysate or sucrose was measured daily after 5 h as described for males. In contrast to males, however, females could mate, and the eggs deposited in the egg-laying devices were collected daily. To allow mating and fertilization of eggs, three adult males which had been kept separately but were of the same age as the females and were well fed with solid protein hydrolysate to assure proper sexual development, were introduced into the individual female cages at the age of 10 days and removed after 5 days, at the age of 15 days old. The males were also removed from the cage for 5 h daily, during the time of capillary feeding by the female, and reintroduced after the capillary was removed and female intake quantified. The same “mating” procedure was repeated between days 30 and 35 of adult age. This experimental design with female medflies allowed us to measure daily intake of the tested solutions and egg production during a long-term period (for more than 40 days in most cases and in several cases for up to 60 days), until all individual females died. Male individual consumption was followed also until the last individual died, which extended for more than 150 days.

### Data analysis and modeling of age related diet intake

Revealing potential interrelationship between medfly diet intake, egg production, and longevity is a complex—even though challenging—task, mainly due to the non-linear nature of the egg production and survivorship curves. Consequently, we used different modeling approaches to get a consensus on their interrelationships. We implemented event history charts, ANOVA and repeated measure analysis, as well as 3D non-parametric and parametric models as described below.

First, the description of daily intake and egg-production throughout the adult life of individual insects was conducted using event history charts (Carey et al., 1998). Event history charts allow plotting the daily consumption or egg-production of the individual fly throughout life, providing a visual method to explore intensity in food consumption and egg-laying as related to adult age.

Second, all data were further evaluated under the assumptions of normality and differences in solution intake between concentrations of protein and sucrose were inferred using typical parametric ANOVA and the non-parametric Kruskal-Wallis method (SPSS, 2013). The effect of adult age, diet type and sex until day 30, as well as their interactions, were inferred using repeated measures ANOVA (Girden, 1992). The between subject effects were tested using Pillai's trace while the assumption of Sphericity was tested using Mauchly's test (*a* = 0.05). Within subject effects was performed when sphericity was not assumed using the Greenhouse and Geisser (1959) and Huynh and Feldt (1976) procedures to correct the degrees of freedom for the *F*-distribution. The effect of diet on medfly longevity and hazard rate was compared using Kaplan Meier survival analysis followed by log-rank tests (*a* = 0.05).

Finally, the relationship between age, dietary intake, and egg production was explored, until all individuals died, using the non-parametric interpolation method LOESS (LOcally WEighted Scatter-plot Smoother, Cleveland, 1979; Cleveland and Devlin, 1988) and a parametric non-linear regression based on a 3D Lorenzian type distribution function (Devanne et al., 2006).

The LOESS procedure allows great flexibility because no assumptions about the parametric form of the regression surface are needed and is suitable when there are outliers in the data and a robust fitting method is necessary (Jacoby, 2000). In brief, the main features of the LOESS procedure assumes that for *i* = 1,….,*n* the *i*th measurement *y*_*i*_ of the response *y* and the corresponding measurement *x*_*i*_ of the vector *x* of *p* predictors are related by: *y*_*i*_ = *g*(*x*_*i*_) + ε_*i*_. Where *g* is a predefined smooth function (i.e., local polynomial) and ε_*i*_ is a random error (Jacoby, 2000). The function *g* can be locally approximated by the value of a regression function, of some specified parametric class and the method can further be extended in the space domain (i.e., 3D, Lekien and Mardsen, 2005). The size of the neighborhoods for local regression is further defined by a smoothing parameter, *q*: (λ + 1)/*n* < *q* < 1. Where λ is the degree of the local polynomial for sample *n* and regressed using a weight function, *w*, to perform a least square fit (SAS Institute Inc., 1999).

In this study, we have used a standard tricube weight function of the form: $w\left(x_{i}\right)={\left(1-{\left|x_{i}\right|}^{3}\right)}^{3}$, for |*x*_*i*_| < 1 and *w*(*x*_*i*_) = 0, for |*x*_*i*_| > 1 and a local polynomial regression of degree 1. Moreover, sampling was performed for a smoothness parameter *q* = 0.1 for the protein intake data and for *q* = 0.2 for the sucrose solutions, respectively. “Lower” parameter values were also tested but proved to be sensitive to extreme sampling error since they pushed the highest point of the 3D mesh and caused in some cases overestimations.

Although the non-parametric interpolation method is very flexible for modeling complex processes, for which no theoretical model exists, it does not provide any abstract information on the significant interaction between variables. Therefore, as a complement, we have decided to run 3D parametric regression mesh model, using ordinal regressions based on a Lorenzian type of normal distribution. The model is given as follows (Devanne et al., 2006):

$$f\left(x,y\right)=\frac{\alpha }{\left[(1+{\left(\frac{x-x0}{\beta }\right)}^{2}]\;\cdot \;[1+{\left(\frac{y-y0}{\gamma }\right)}^{2}\right]}\cdot$$

The above function has five parameters *x*0, *y*0, α, β, and γ, and which can be interpreted to a certain degree. Parameter α represents the estimated amplitude of the maximal egg production that is located at coordinates *x*0 and *y*0. Moreover, β and γ reflect the slope and width of the egg production peak in relation to diet intake and age, respectively.

The slope parameters α and β obtained from the Lorentzian function, as well as the differences between the coordinates (*x0, y0*), were compared using one-way ANOVA and the Student's *t*-test for 0.05 significance level. The interpolation models were generated using Macro Recorder script modules and the Interactive Development Environment (IDE) in SigmaPlot (SigmaPlot® 8.0 Programming Guide)^1^.

## Results

### Standardization of the methodology

**Figure 2:**
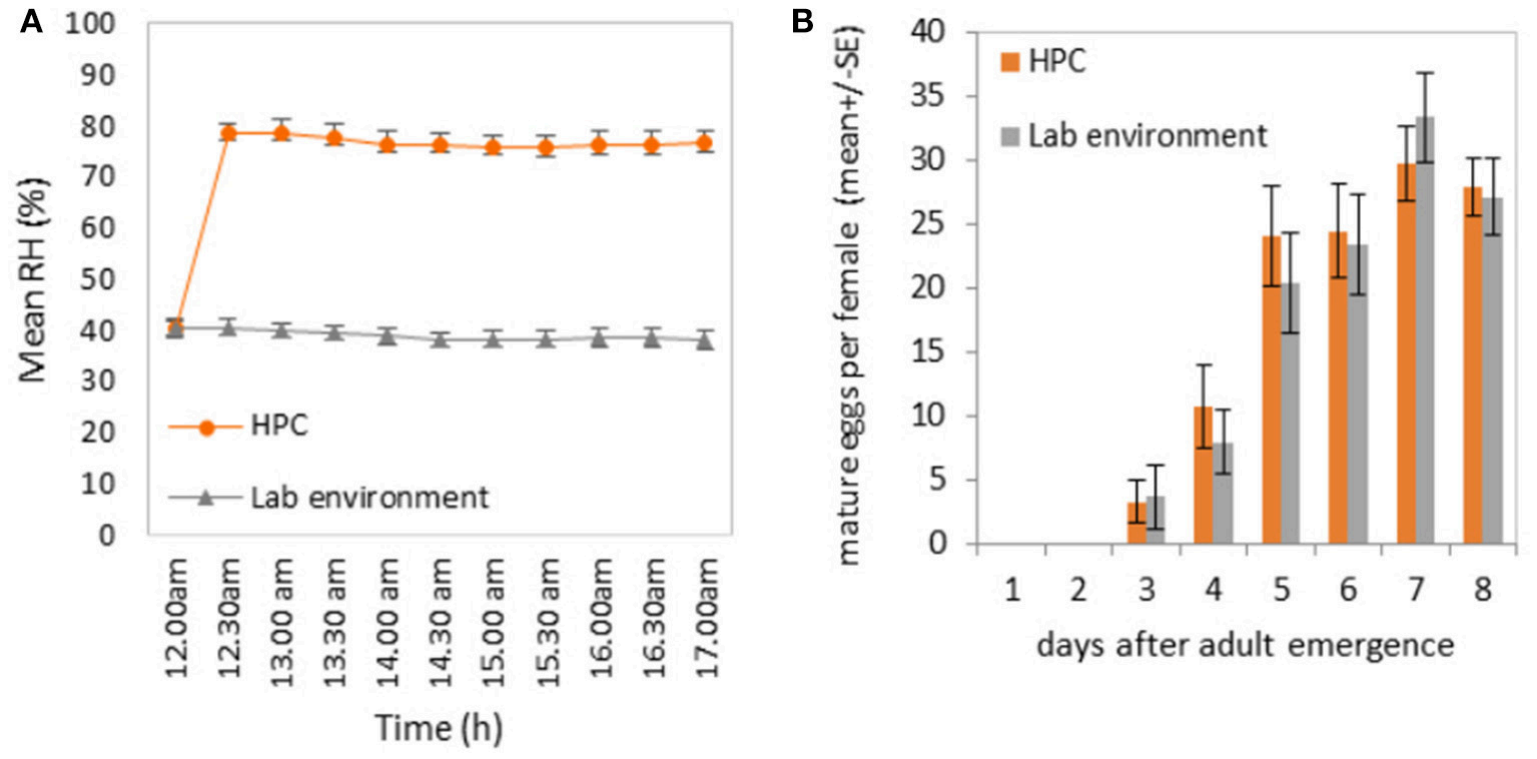
**(A)** Relative humidity (RH) levels inside the “Humidity Preservation Chambers” (HPC) and in the laboratory environment (25 ± 2°C). Measures were performed every half hour for a total of 5 h (from 12:00 to 17:00). **(B)** Effect of the two relative humidity environments on the number of ovarioles per fruit fly.

Relative humidity inside the HPC strongly contrasted with the relative humidity measured in the control cages and in the laboratory environment (Figure 2A). While the relative humidity inside the HPC was kept around 80% during the afternoon hours (12:00–17:00 h), the ambient relative humidity in the laboratory during that period of the day was around 40%. The effect of the two different humidity environments did not affect the overall egg-laying potential, as reflected by the similarities in oogenesis of female medflies in the two types of environments (Figure 2B; *t* = 0.436, *df* = 38, *p* = 0.66). These results strengthened the use of the HPC to constantly maintain a high level of humidity during the long-term food-intake and egg-production study, thus avoiding undesirable evaporation of the liquid food.

**Figure 3:**
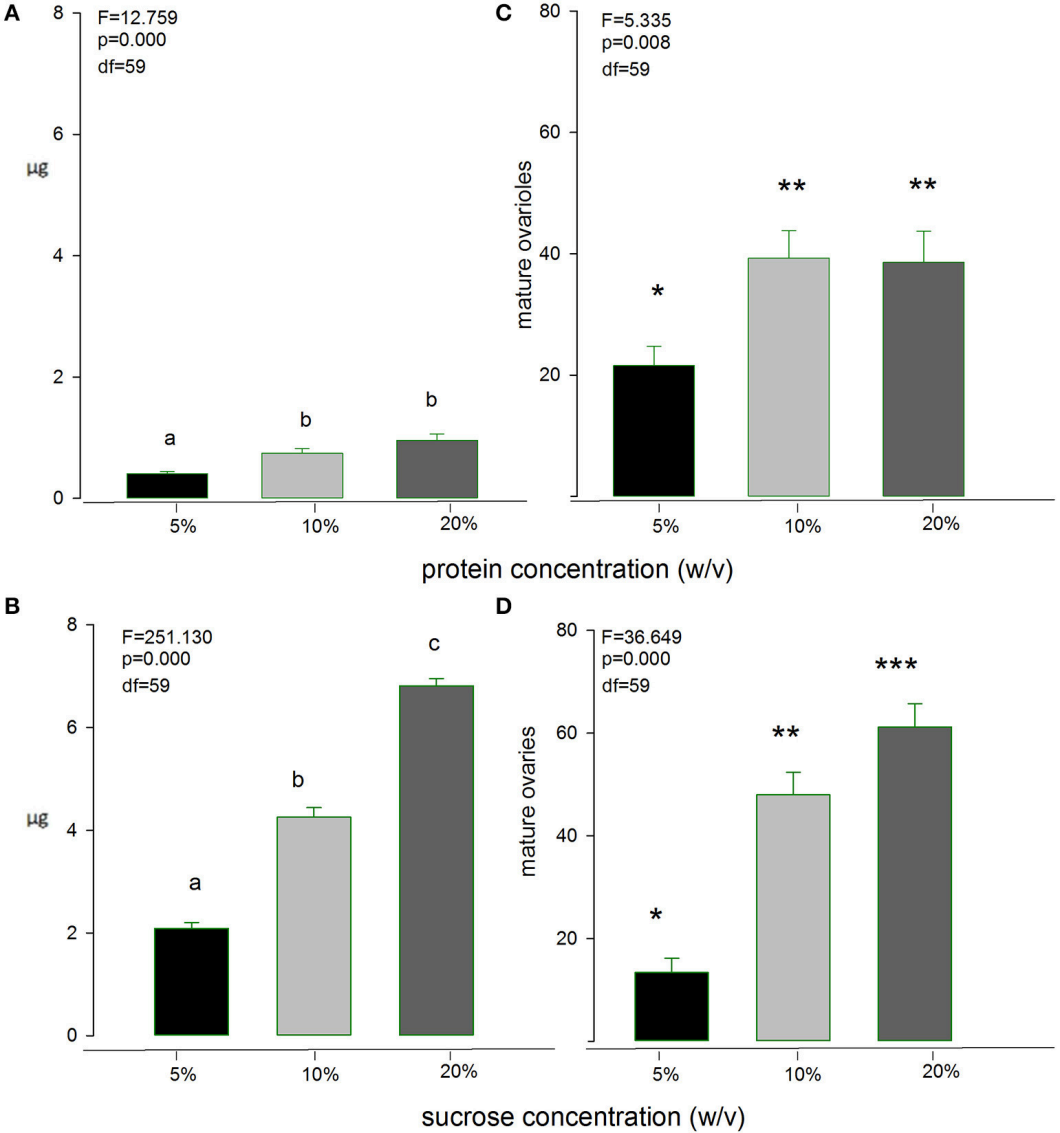
**Intake of Protein-hydrolysate solution (A)** and sugar **(B)** by medfly females as affected by the concentration of the solution, and effect of solution concentration on maturation of ovarioles **(C,D)**. Bars with different symbols or letters are significantly different at 0.05 level.

An important aspect of our study was to estimate the “optimal” concentration of nutrient solutions that will maintain good levels of nutrition and egg-production in female medflies, but will also allow quantifying nutrient uptake with a large degree of certainty and reliability. Figure 3 shows both the cumulative amount of nutrient ingested during 15 days by individual females as affected by the concentration of sucrose and protein hydrolysate solutions and the effect of food concentration on the number of mature ovarioles developed by single females during the 15 days of the experiment. Both protein hydrolysate and sucrose ingested amounts were significantly larger in the 20% solutions than in the others (*F* = 12.76, *df* = 59, *p* > 0.01 and *F* = 251.13, *df* = 59, *p* < 0.01 for sucrose and protein hydrolysate solutions, respectively), and the number of ovarioles was significantly favored by the 20% solutions of both nutrients (*F* = 5.34, *df* = 59, *p* < 0.01 and *F* = 38.65, *df* = 59, *p* < 0.01 for sucrose and protein hydrolysate solutions, respectively). These results, thus, pointed at the 20% solutions as appropriate for the long-term intake and egg-laying study.

### Patterns of nutrient uptake by male and female medflies and female Egg-laying

**Figure 4:**
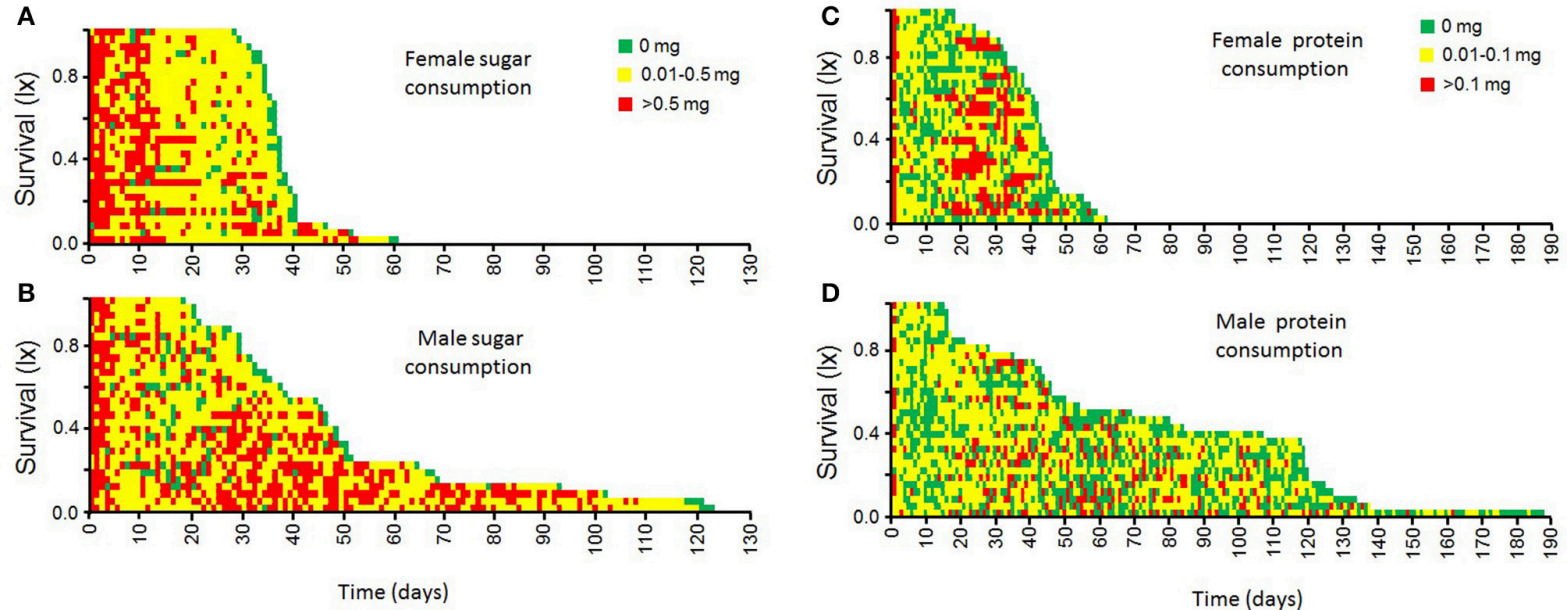
**Individual patterns of sucrose (A,B)** and protein hydrolysate **(C,D)** consumption by female and male medflies throughout their life.

Age-specific intake of nutrients by adult female and male medfly is shown in the event history charts in Figure 4. Both, males and females, ingest relatively large amounts of sucrose during the first days of adult-life (first 10 days). Sucrose consumption in females then drops and is maintained relatively low until death. Males, in contrast, show an intermittent consumption of sucrose solutions, which is marked by a drop in adult ages 10–20 days, and increased sucrose intake in mid-life and advanced ages (30–100 days old).

**Figure 5:**
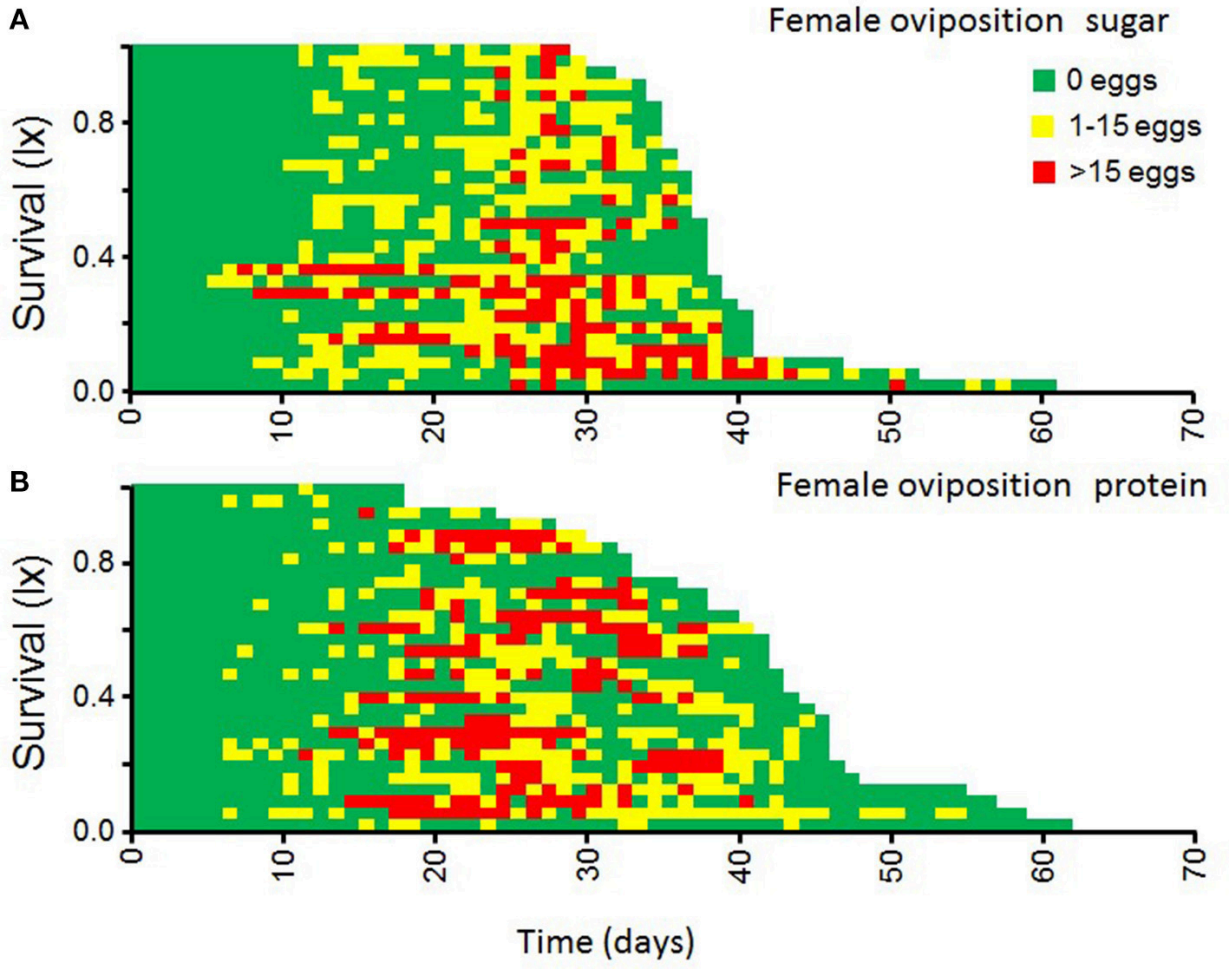
**Patterns of individual egg-laying in female medflies fed sucrose solutions (A)** and protein **(B)** hydrolysate solutions.

Female medflies tend to have an initial relatively large intake of protein hydrolysate during the first 2 days of adult life, then it drops and increases toward mid-life (20–40 days old), when the first batch of eggs are being laid (Figures 4, 5). Males, in contrast, showed a more stable and constant intake of moderate levels of protein hydrolysate throughout their adult life-span (Figure 4).

Egg-laying patterns as affected by the solution of nutrient provided to the female are shown in Figure 5. Females fed with protein solutions produced similar numbers of eggs per day compared to females fed with sucrose solutions. The main wave of egg production coincides with the patterns of intake of protein hydrolysate solution by female flies (Figure 4). Apparently, diet type did not significantly affect the life time egg production (*F* = 1.63, *df* = 1, *p* = 0.21).

**Figure 6:**
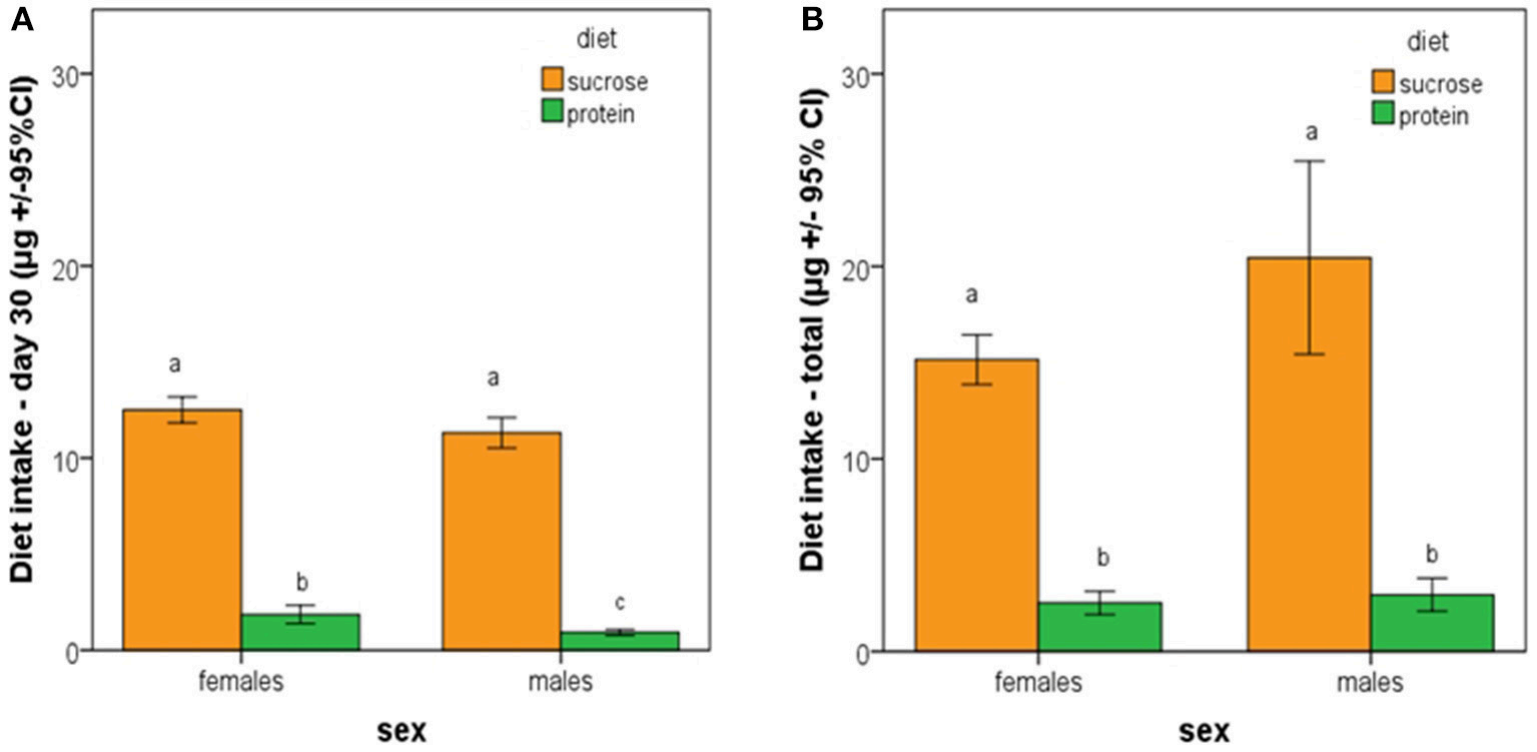
**Average individual cumulative ingestion of sucrose and protein hydrolysate by female and male medflies during (A)** the first 30 days of adult life and **(B)** during total adult life. Bars with different letters are significantly different at 0.05 level.

Average individual intake of sucrose during the first 30 days did not differ between male and female medflies. In contrast, individual intake of protein hydrolysate during the first 30 days of adult life was significantly higher in female than in male medflies. Both males and females consumed significant higher amounts of sucrose than protein hydrolysate (Figure 6A; *F* = 469.972, *df* = 119, *p* < 0.005). Total nutrient consumption by males and females fed with sucrose or protein hydrolysate throughout their lifespan was also compared and it was found that it did not differ between male and female medflies (*p* > 0.05). Additionally, both sexes consumed significant higher amounts of sucrose than protein hydrolysate (Figure 6B; *F* = 48.217, *df* = 119, *p* < 0.005).

The Log Rank (Mantel-Cox) test revealed significant differences among the four adult cohorts (*X*^2^ = 29.128, *df* = 3, and *p* < 0.005). Particularly, male longevity was longer than that of females (Table 1). Furthermore, the ingestion of protein hydrolysate solutions significantly expanded longevity, at least in male medflies.

**Table 1:** **Survival time of male and female medfly cohorts fed with sugars and proteins**.

**Diet**	**Gender**	**Mean**			**Median**		
		**Estimate ± *SE***	**95% confidence interval**		**Estimate ± *SE***	**95% confidence interval**	
			**CI low**	**CI high**		**CI low**	**CI high**
Sugar	Female	38.935 ± 1.133	36.715	41.156	38.000 ± 0.556	36.909	39.091
	Male	50.833 ± 5.031	40.973	60.694	46.000 ± 4.930	36.338	55.662
Protein	Female	40.057 ± 1.925	36.285	43.829	43.000 ± 1.932	39.213	46.787
	Male	75.037 ± 9.296	56.817	93.257	55.000 ± 19.905	15.986	94.014
Overall		50.081 ± 2.738	44.715	55.448	42.000 ± 1.201	39.647	44.353

**Figure 7:**
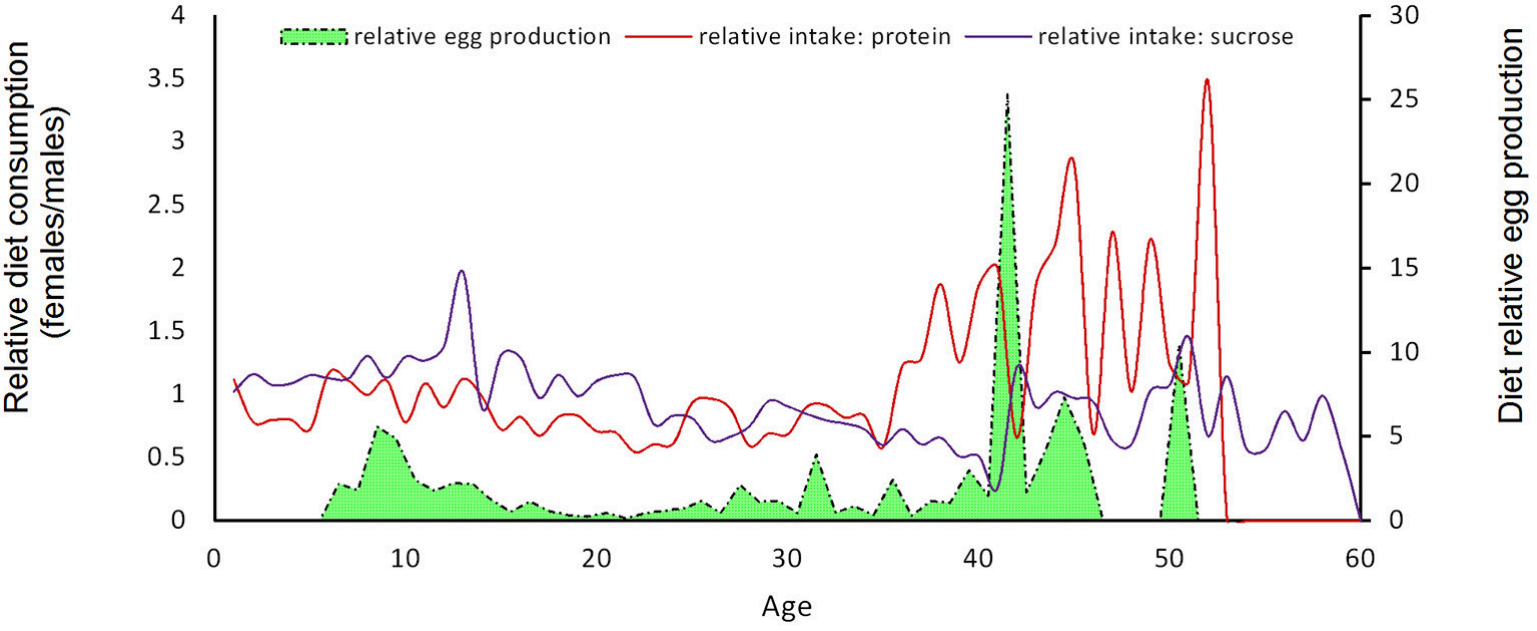
**Female/male medfly relative diet ingestion of sugars and proteins (in μg) until day 60 and relative egg production (eggs with sugar intake/eggs with protein intake)**.

Patterns in nutrient consumption by males and females throughout their lifespan were explored by contrasting the average consumption between the two sexes. Figure 7 shows the relative female/male diet consumption of protein hydrolyzate and sucrose until day 60 of adult life. During the first 30 days, consumption of sucrose and protein hydrolyzate is relatively similar in the two sexes. However, after day 30 of adult life, females tend to double, and even triplicate, the ingestion of protein compared to males (Figure 7). Consumption of protein by females oscillates in time with peaks of consumption (of up to 3.5 times that observed in males) with a periodicity of a few days between peaks. Consumption of the sugar solution after day 30 is kept in average at around 1, with slight oscillations showing higher consumption of sugar by male medflies. However, correlations between relative diet consumptions and egg productions for individuals feed with sucrose or protein solution were not significant (*p* > 0.05).

### Non-linear modeling Egg-laying as a function of nutrient uptake and age

**Figure 8:**
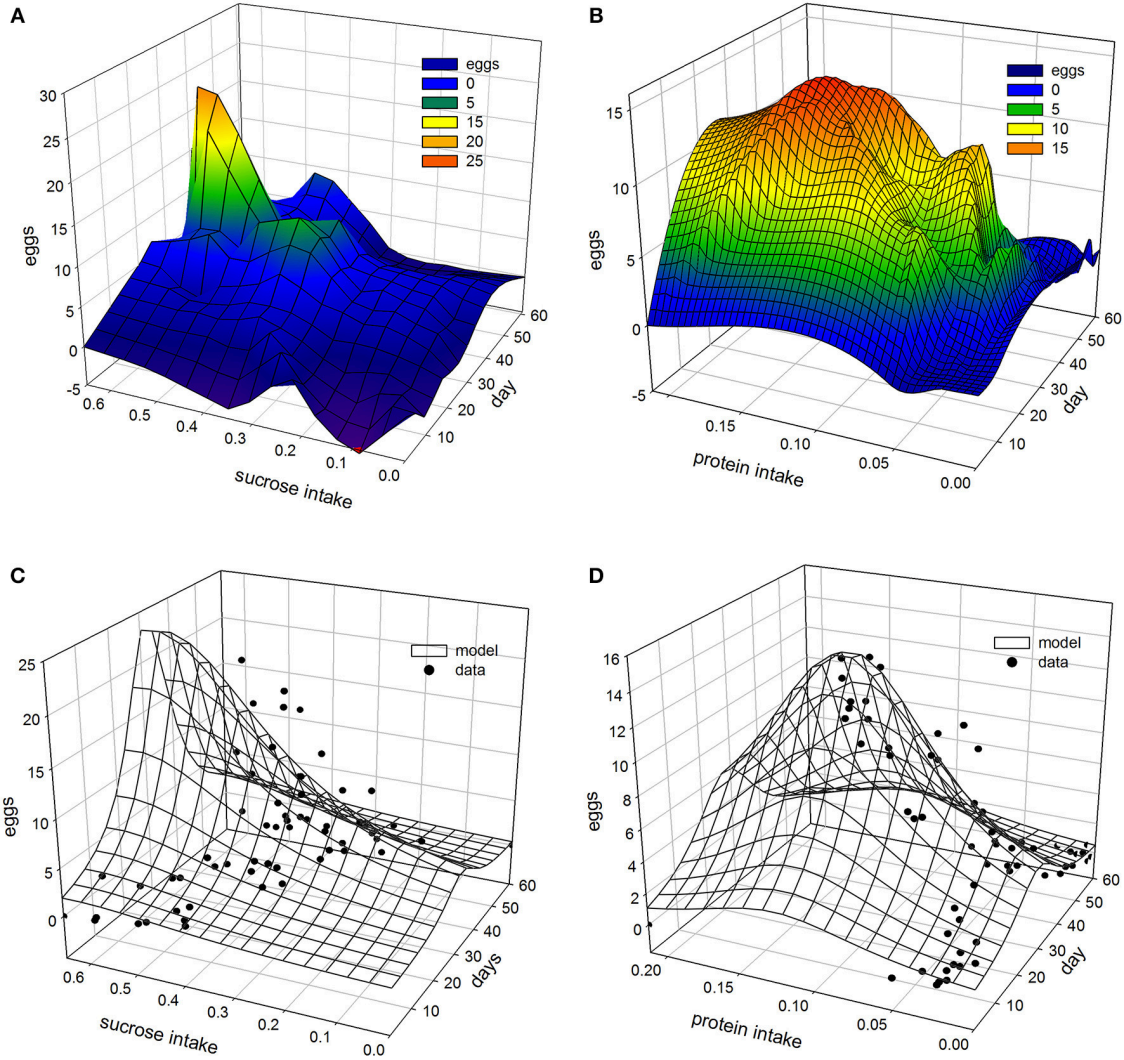
**Egg production as a function of diet intake using 3 days interpolation LOESS method for sugar (A)** and protein **(B)** and 3D Lorentzian regression model for sugar **(C)** and protein **(D)**.

Non-linear modeling provides a surface-plot analysis methodology to link intake with egg-production and age, which is not possible using other types of analysis. Age related egg-laying progression differs between medflies fed with sucrose and protein hydrolysate (Figures 8A,B). Large number of laid eggs is related both to a relatively large consumption of sucrose and a relatively intermediate intake level of proteins. The highest level of egg production was observed at the age of 40–50 days when medflies consumed between 0.5 and 0.6 μg sucrose and between 0.12 and 0.17 μg of protein hydrolysate. Egg-laying, thus, is maximal at mid-life of the cohorts and appears to be bell shaped with pronounced tails. Moreover, fitting of the Lorentzian function to the data points allowed calculation of the coordinates of the optimal point of egg production (i.e., parameters *x0* and *y0*). Additionally, the two slope parameters, α and β, which give an indication of the shape of data distribution were significant in most cases, although the coefficient of determination was lower (0.489) for the sucrose egg production data compared to the protein (0.903). Moreover, the respective ANOVA model accounted for most of the data variability and *F*-statistic was significant for both the sucrose egg production data (*F* = 13.205, *df* = 59, *p* < 0.000) and the protein data (*F* = 129.401, *df* = 59, *p* < 0.000), respectively.

Consequently, both patterns could be described well by the Lorenzian type regression functions. In addition, the regression models for all variables were found to be significant (Tables 2, 3). Figures 8C,D show the fitting of the data to the models. Indeed, egg production data, and especially that related to protein hydrolysate intake, were soundly described by the Lorenzian type normal distribution function, with 95% of the variability accounted by the data (Tables 2, 3). The parameter terms were found significant in most cases (*p* < 0.05) and their influence was found to be the most important in ANOVA model calculations.

**Table 2A:** **Non-linear 3D model regression statistics to describe the relation between sugar intake, egg production, and age in the medfly**.

**Model**	***R***	***R*^2^**	**Adjusted *R*^2^**	***SE* of estimate**
3D, Lorentzian	0.6999	0.4899	0.4528	3.7487

**Table 2B:** **Model parameters**.

**Coefficient**		***SE***	***t***	***p*-value**
*x*0	32.0000	1.1310	28.2947	<0.0001
*y*0	0.6276	0.2476	2.5341	0.0142
α	24.0087	17.9864	1.3348	0.1874
β	9.2432	1.7346	5.3288	<0.0001
γ	0.2749	0.1077	2.5531	0.0135

**Table 2C:** **Analysis of variance: corrected for the mean of the observations**.

	***df***	**SS**	**MS**	***F***	***p*-value**
Regression	4	742.2742	185.5686	13.2055	<0.0001
Residual	55	772.8826	14.0524		
Total	59	1515.1569	25.6806		

**Table 3A:** **Non-linear 3D model regression statistics to describe the relation between protein intake, egg production, and age in the medfly**.

**Model**	***R***	***R^2^***	**Adjusted *R^2^***	***SE* of estimate**
3D, Lorentzian	0.9508	0.9039	0.8970	1.5619

**Table 3B:** **Model parameters**.

**Coefficient**		***SE***	***t***	***p*-value**
*x*0	28.3359	0.6892	41.1140	<0.0001
*y*0	0.1258	0.0134	9.3826	<0.0001
α	14.8297	1.0309	14.3857	<0.0001
β	12.7151	1.4940	8.5106	<0.0001
γ	0.0725	0.0137	5.2809	<0.0001

**Table 3C:** **Analysis of variance: corrected for the mean of the observations**.

	***df***	**SS**	**MS**	***F***	***p*-value**
Regression	4	1262.7094	315.6773	129.4014	<0.0001
Residual	55	134.1736	2.4395		
Total	59	1396.8830	23.6760		

## Discussion

In this study, we developed and standardized a methodology suitable for measuring individual daily food ingestion of the adult *C. capitata* throughout life span. This method enables high precision measuring of daily food intake while at the same time allows to relate intake to individual egg production in fruit flies, which has rarely been concomitantly measured. Moreover, in contrast to other studies, which explore diet consumption during a short period of the insect's life, the current work screens diet consumption until all individuals have died. This is a major advantage because the influence of nutrition may affect not only the metabolic needs and reproductive potential during early ages but also have differential effects throughout the entire lifespan.

One important novelty of the current experimental set up, compared to other studies, is that medflies had *ad libitum* access to solid diet while presented to a liquid solution of food. This exposure of flies to two different sources of food allowed us to explore intake of one of the nutritional food sources in the presence of an alternative food source. This provides information on the timing and need of specific nutritional elements during development and lifespan of the insects. For example, the results, confirmed that *ad libitum* laboratory reared medfly has an ability to consume high energy sources in the form of solutions during a range of its lifespan which coincides with a high reproductive budged. In fact, this type of experimental setting could provide an elegant framework to investigate the prandiology of specific nutrients, such as single amino acids, or the phagostimulatory properties, and reproductive effects, of secondary plant metabolites or other ingested substances, such as the parapheromone methyl eugenol (Tan et al., 2014).

The CAFE capillary method developed by Ja et al. (2007) for *Drosophila*, despite being an effective system to measure intake, may substantially reduce egg-laying and life span compared to techniques which provide food in agar-gelled medium (Bass et al., 2007; Ja et al., 2007; Wong et al., 2009). In the case of our study, with the modified CAFE system, egg-laying and life-span were comparable to studies using the provision of add-libitum food sources (Carey, 2003). Moreover, compared to indirect measures, which are usually based on regular administration of radioactively-labeled nutrients after transferring flies to food (King and Wilson, 1955; Carvalho et al., 2005), our system provides undisturbed feeding conditions and allows measures of intake and egg-laying for long-periods of time without possible adverse effects on reproduction.

A limitation of our method is that when liquid diet is provided, older adults may not be able to have free access to the food solution provided in capillaries positioned in the roof of the individual cages. As we have shown in an earlier study adult medflies at older ages lose capacity to climb on cage walls and spend a progressively increasing amount of time in a supine position (Papadopoulos et al., 2002). Apparently, these senescent adults have limited food location capacity. Nonetheless, this does not affect both conclusions regarding food consumption during the active life span of the individuals and the aim of the current paper, which was to demonstrate and test a prandiology system for life time assessment of food intake. Simple adjustments, regarding positioning of the capillaries within the individual cage, may address this issue. It should also be noted that liquid diet influences food intake and energy balance in an organism in a very different and sometimes complex way relative to a comparable solid diet (Simpson and Raubenheimer, 1999; DiMeglio and Mattes, 2000). This is further supported by the burst in egg production observed in our study after day 40 in females eating less sugar, but more protein (see Figure 7). Even though no statistically significantly correlation exists, this outcome supports the notion of protein as a booster of egg production and the possibility that protein provided in liquid form enables a higher egg-production yield than solid protein provided *ad libitum*. We therefore suggest that researchers with different objectives than ours should consider providing food in the same form, either only liquid or only solid.

Our results suggest that the high relative humidity (i.e., HPC vs. the lab environment) did not affect the overall egg-laying potential, as reflected by the similarities in the number of ovarioles in female ovaries. In general, tephritids are mainly tropical or subtropical in origin and need hot, damp (50–80% relative humidity) conditions for optimum egg production (Fay, 1989). For example, in *Bactrocera oleae* low relative humidity suppressed ovarian maturation, egg production, and longevity whereas high relative humidity in the range from 55 to 75% favored them (Broufas et al., 2009).

Regardless of diet type males in this experiment survived significantly longer than females. Similar results have been reported by Diamantidis et al. (2009) testing six different medfly biotypes. As far as the effects of protein availability on male and female longevity are concerned, the results are rather controversial. For example, Plácido-Silva et al. (2006) and Chang et al. (2001), did not observe any differences between males and females when fed with similar amounts of protein diet. However, in a seminal paper, Muller et al. (1997) have proven that life expectancy of both male and female medflies is reduced if they are deprived from protein and that the reduction is far greater in females resulting in a reversal of female advantage in life expectancy. It seems that external factors, such as dietary manipulations may alter life span advantage of the one over the other sex (Carey, 2003).

In the current experiments females were mated but males were not. Mating is known to induce cost in terms of life span reduction in both female and male medflies (Papadopoulos et al., 2010; Papanastasiou et al., 2013). Similar results regarding the cost of mating have also been reported for the olive fly (Gerofotis et al., 2015). This lack of mating cost may in part account for the substantially larger lifespan in males relative to females observed in our study. It is well appreciated in fruit fly research that females need high amounts of protein for egg laying and apparently sugar to fulfill energy requirements induced by other behavioral activities. Energy requirements are minimal in the small individual cages since foraging demands (search for oviposition sites and food) are essentially absent. On the other hand, it has been shown recently that male medflies require increased amounts of both sugar and protein intake to perform the various energetically demanding sexual calling activities of pheromone production, wing vibration, etc. (Papadopoulos et al., 1998; Papanastasiou et al., 2013). Female egg laying and male sexual activities are both energetically and dietary demanding and may thus trade-off with lifespan in ways that result in differences between the two sexes. In our case, differences are more profound in males because the overall energy taken from food can be partitioned only in lifespan, given that males in individual cages were prevented from any other energetically costly activity (e.g., aggressive behavior to other rivals). In females, the overall energy was partitioned between lifespan and egg-laying and thus the magnitude of the effects on each trait is expected to be different than in males and much harder to detect. However, the current experimental set up does not test for possible trade-offs and neither dietary nor calorie restriction or other effects.

Regarding the different modeling approaches, which were applied to the data, it is important to outline that each has specific advantages and limitations and thus were all used to find a consensus on the interrelations between dietary intake, egg production and longevity. The event history charts, for example, have the advantage of discovering a specific behavioral pattern by age, yet are mostly descriptive. Furthermore, the use of typical statistical models, such as one-way ANOVA, does not account for the effects of age in the analysis (Field, 2011). On the other hand, the repeated measures analysis, although it provides a good way of statistically assessing the change of a variable over time, it is strongly affected when within subject factors exceed more than two levels and is vulnerable to effects from missing values (i.e., mortality at advantaged ages). In respect to the non-parametric LOESS method, the biggest advantage is that it does not require the specification of a function to fit a model to all the sample data and the analyst must provide simply the degree of polynomial and smoothing parameter value. However, it's rather difficult to statistically evaluate the functional dependence between the variables of interest. Ultimately, the parametric 3D surface models may be viewed as a way of merging the observed actual event history data of a cohort with a statistical model, although there are cases where it's quite difficult to obtain a suitable model that fits over all data set. For example, the Lorentzian like model fits remarkably better to the protein data compared to the sucrose data density in which both slope parameters, α and β, where significant and gave an indication of the egg production steepness of the 3D surfaces in respect to diet intake and aging.

Thus, the 3D interpolation models produced good estimates of egg production and diet intake as affected by age, providing us with a reliable multivariate analytical tool to model nutritional trends in insects, and other organisms, and their effect upon life history traits. To our knowledge, this modeling has seldom been used to relate individual fruit fly intake with egg-laying, providing us with an excellent approach in prandiology studies, not only of insects but for other organisms as well. This type of modeling allowed us to strengthen the knowledge that egg production is closely related to diet consumption, as suggested by the shape of the medfly reproduction-response function and its functional relationship to diet intake and age. It also provided day by day information of this biological relationship. We believe that its use may allow more insights into the functional relationships between nutrition and other biological traits in higher organisms.

## Author contributions

NK, NP, DN, and CI conceived and designed experiments; CT and CI performed experiments; PD, CT, NK, and CI prepared figures and tables; PD, CI, DN, NK, NP, and DK analyzed and interpreted results, and wrote the paper; NK edited and revised the manuscript.

### Conflict of interest statement

The authors declare that the research was conducted in the absence of any commercial or financial relationships that could be construed as a potential conflict of interest. The reviewers, JJS and SR, and handling Editor declared their shared affiliation, and the handling Editor states that the process nevertheless met the standards of a fair and objective review.

## Footnotes

^1^SigmaPlot® 8.0. Programming Guide. Copyright © 2002 by SPSS Inc.
